# Long-term metformin treatment in adolescents with obesity and insulin resistance, results of an open label extension study

**DOI:** 10.1038/s41387-018-0057-6

**Published:** 2018-09-10

**Authors:** Y. E. Lentferink, M. P. van der Aa, E. G. A. H. van Mill, C. A. J. Knibbe, M. M. J. van der Vorst

**Affiliations:** 1 Department of Pediatrics, St. Antonius Hospital, Nieuwegein/Utrecht, The Netherlands; 2 Department of Pediatrics, Jeroen Bosch Hospital, ‘s-Hertogenbosch, The Netherlands; 3 Department of Clinical Pharmacy, St. Antonius Hospital, Nieuwegein/Utrecht, The Netherlands; 4 Division of Pharmacology, LACDR, Leiden University, Leiden, The Netherlands; 5 Present Address: Kalkhaven, Gorinchem, The Netherlands

## Abstract

**Background/Objectives:**

Off-label metformin is nowadays frequently used for the treatment of obesity in adolescents. However, studies on long-term metformin treatment in adolescents with obesity are scarce. Therefore, an 18 month open label extension study following an 18 months randomized placebo-controlled trial (RCT) on the efficacy, safety, and tolerability of metformin in adolescents with obesity and insulin resistance was performed.

**Subjects/Methods:**

After completion of the RCT, metformin was offered to all participants with a body mass index standard deviation score (BMI-sds) > 2.3 and Homeostasis Model Assessment for Insulin Resistance (HOMA-IR) ≥ 3.4. Endpoints were change in BMI and HOMA-IR.

**Results:**

Overall, 31/42 participants completed the extension study (74% girls, median age 14.8 (11.6 – 17.9), BMI 31.2 (22.3 – 45.1), HOMA-IR 3.4 (0.2 – 8.8)). At start, 22/42 (52.4%) participants were eligible for metformin of which 13 (59.0%) agreed with treatment. In participants who continued metformin, an increase was observed in BMI (+2.2 (+0.2 to +9.0)) and HOMA-IR (+13.7 (+1.6 to +48.3)). In metformin naive participants, BMI stabilized after an initial decrease (+0.5 (−2.1 to +5.1)). For HOMA-IR, a decrease was observed (−1.1 (−4.6 to +1.4)).

**Conclusion:**

While metformin treatment in metformin naive participants seems to result in an initial decrease in BMI and HOMA-IR, there is no evidence for sustained effect after prolonged use in adolescents. Limited compliance and/or insufficient dose may explain the differences in long-term effects between adolescents and adults.

## Introduction

Obesity is a major health problem worldwide^1^, with an estimated prevalence in children and adolescents up to 5.4% in 2025^2^. Obesity is associated with complications such as the metabolic syndrome, type 2 diabetes mellitus (T2DM), cardiovascular diseases, and hepatic steatosis^2,3^. Insulin resistance (IR) has an important role in the development of complications, as it is the precursor of a disturbed glucose tolerance^4,5^, recognized as an independent risk factor for cardiovascular diseases^4,^ and part of the metabolic syndrome^5,6^. Since obesity is moving towards a younger age, related complications will become manifest during childhood^3^. Multidisciplinary lifestyle intervention is the cornerstone of (pediatric) obesity treatment^7^. However, it is associated with only a marginal long-term effect due to high dropout rates and limited motivation observed in nearly all studies^7,8^.

In recent years, studies are focusing on additional therapies on top of lifestyle intervention such as pharmacotherapy and bariatric surgery^7^. Bariatric surgery, although not yet considered as standard therapy, is suggested to be effective in postpubertal adolescents with therapy-resistant obesity^9–11^. Concerning pharmacotherapy, orlistat and metformin are the two most studied drugs^7,12^. Orlistat, a lipase inhibitor, is the only approved drug for the treatment of childhood obesity. However, usefulness in daily clinical practice is limited due to frequently reported gastrointestinal adverse effects and only modest decrease in weight without beneficial effects on cardiometabolic complication^7,12–14^.

Metformin, an oral antihyperglycemic agent approved for the treatment of T2DM from the age of 10 years onwards, has been the focus of multiple trials as additional therapy in the treatment of pediatric obesity^15–24^. It is associated with small but significant reductions in weight and generally well-tolerated^15–24^. Although literature is inconsistent, favorable effects of metformin on cardiometabolic complications have been described^15–18,21^. Therefore, it is suggested that metformin could have potentials in delaying and/or preventing complications of (pediatric) obesity^25^. Studies on the efficacy of metformin in adolescents are however predominantly limited to a follow-up period of 6 months^15–20^. Only a few studies have been performed with a longer follow-up period with a maximum up to 24 months^21–24^. Consequently, it is unclear whether prolonged metformin treatment in adolescents will result in long-lasting positive effects on weight.

Therefore, the aim of this study is to report on the results of an 18 months open label extension study following a randomized placebo-controlled trial (RCT) on 18 months treatment with metformin or placebo with respect to efficacy, safety, and tolerability of metformin treatment in adolescents with obesity and IR^22^. Moreover, the development of obesity-related metabolic and cardiovascular complications are evaluated.

## Materials and methods

Since the trial protocol and the results of the 18 months RCT have been reported elsewhere^22,26^, only a brief description of the study design is presented here.

### Study design and participants

This study is an 18 month open label extension study following the RCT on 18 months treatment with metformin in adolescents with obesity and IR^22^, which was performed in the St. Antonius Hospital Nieuwegein/Utrecht and Jeroen Bosch Hospital ‘s Hertogenbosch, the Netherlands (ClinicalTrials.gov number NCT01487993). The study protocol was approved by the Medical Ethical Committee of the St. Antonius Hospital, Nieuwegein/Utrecht, the Netherlands. From all participants/parents a written informed consent was obtained at start RCT. All study procedures were in accordance with the Declaration of Helsinki and the Medical Research Involving Human Subjects Act (WMO) of the Netherlands. All participants who completed the RCT, irrespective of obesity and IR status, were included in this open label extension study (July 2013–February 2017). At start of the open label extension study, participants were not informed in which treatment arm they were allocated, since the randomization code was only opened after the last participant, included in the RCT had finished the RCT. Metformin therapy was only offered to participants who still suffered from obesity (defined as body mass index standard deviation score (BMI-sds > 2.3))^27^, and IR (defined as Homeostasis Model Assessment for Insulin Resistance (HOMA-IR) ≥ 3.4). Participants not meeting these criteria or disagree with metformin treatment, did not use metformin during the extension study.

Consequently, there were four study-arms in this open label extension study depending on the use of metformin or placebo during the RCT and metformin treatment in this study. Participants with metformin during the open label extension study were labeled MM or PM, participants without metformin were labeled MP or PP. The first letter represents the treatment during the RCT (M for metformin, P for placebo), the second letter the treatment during the open label extension study.

Measurements were performed at the pediatric outpatient clinics or day-care wards of the participating hospitals. All participants had three scheduled hospital visits and three telephone calls, except metformin users who had additional visits instead of telephone calls to monitor safety and tolerability. The fitness tests were performed at the physical therapy outpatient clinic of the St. Antonius Hospital and at the Sports Medical Centre of the Jeroen Bosch Hospital. In contrast to the RCT no specific supervised physical training program was offered. Similar to the RCT, participants on metformin therapy received immediate-release metformin 500 mg tablets in an increasing dosing regimen, with a maximum of two tablets twice daily in the fourth week. In case of gastrointestinal complaints, the dosage was reduced to the last well-tolerated dose. After symptoms had ceased, the dosage was increased to the maximum dosage tolerated^26^. In contrast to the RCT, no pill counts were performed.

### Outcomes

The endpoints were change in BMI (ΔBMI) and change in HOMA-IR (ΔHOMA-IR). Furthermore, safety and tolerability of metformin were evaluated. In addition, change in HbA1c, body fat percentage, quality of life, and physical fitness were assessed. Lastly, the percentage of obesity-related metabolic and cardiovascular complications was evaluated.

BMI was calculated as weight (kg)/(height (m))^2^. The corresponding age and sex adjusted BMI, the BMI-sds was calculated using the TNO growth calculator for professionals^28^. IR was calculated using HOMA-IR (Fasting Plasma Glucose (mmol/l) × Fasting Plasma Insulin (mU/l))/22.5))^29^, and was defined as a HOMA-IR ≥ 3.4^30^.

Safety was reported as the number of cases with hepatic and/or renal function tests exceeding safety limits (ALAT > 69U/l (girls) or >78 U/l (boys), glomerular filtration rate (GFR) < 60 ml/min) and vitamin B12 deficiency (vitamin B12 < 140 pmol/l). Tolerability was reported as adverse drugs effects in relation with actual metformin dosage.

The body fat percentage was measured by bio-impedance analysis using a Tanita BC-420MA body composition analyzer (Tanita Corporation, Tokyo, Japan). Quality of life was measured with the Impact of Weight on Quality of Life-kids (IWQOL-kids). Physical fitness was evaluated using the modified Shuttle walking test for endurance, while static and dynamic balance test, according to Movement ABC were used to test coordination and strength^26^.

Definitions of metabolic complications: disturbed glucose tolerance was defined as an impaired fasting glucose (IFG) ≥ 5.6–< 7.0 mmol/l, or impaired glucose tolerance (IGT) ≥ 7.8–< 11.1 mmol/l, or T2DM (fasting plasma glucose ≥ 7.0 mmol/l, or 2 h glucose ≥ 11.1 mmol/l)^31^. A high triglyceride was defined as ≥ 1.7 mmol/l and a low high-density lipoprotein (HDL) as < 1.03 mmol/l^31^. A high systolic blood pressure was defined as systolic and/or diastolic blood pressure ≥ 95th percentile for age sex, and height^32^. The metabolic syndrome was defined as the presence of at least 3 of the following criteria: waist circumference ≥ 95th percentile for age, systolic and/or diastolic blood pressure ≥ 95th percentile for age, high triglycerides, low HDL, and disturbed glucose tolerance^6^. Microvascular complications were defined as urine albumin 30-300 mg/l in an early morning urine sample^31^. Cardiovascular complications were evaluated by the arterial stiffness. Arterial stiffness was assessed non-invasively by measuring the pulse wave velocity (PWV) and augmentation index (AIX), using the SphygmoCor (Model SCOR-Px, Software version, 7.01; AtCor Medical Pvt. Ltd, Sydney, Australia).

Anthropometric and laboratory parameters were assessed every 6 months. The body fat percentage, IWQOL-kids, physical fitness test, and arterial stiffness were only assessed at the end of the extension study, therefore data obtained at the end of the RCT were used for comparison.

### Statistical analysis

Statistical analysis was performed using IBM SPSS Statistics, version 24 (IBM SPSS Statistics, Chicago, IL, USA). Since the number of participants per study-arm were small results were presented descriptively, however *p*-values are depicted in the tables. Because of the small sample size the parameters were assumed not to be normally distributed and therefore continuous data were reported as median with range and categorical data as frequencies with percentage. The Kruskal–Wallis test was used to compare baseline characteristics of the study-arms of continuous data and the *χ*^2^ test or Fisher’s exact test for categorical variables. The outcome parameters at time points *t* = 0 and *t* = 18 of the open label extension study were compared for the study-arms using the Kruskal–Wallis test. An *α*-level of 5% was considered significant for all statistical tests.

## Results

**Fig. 1:**
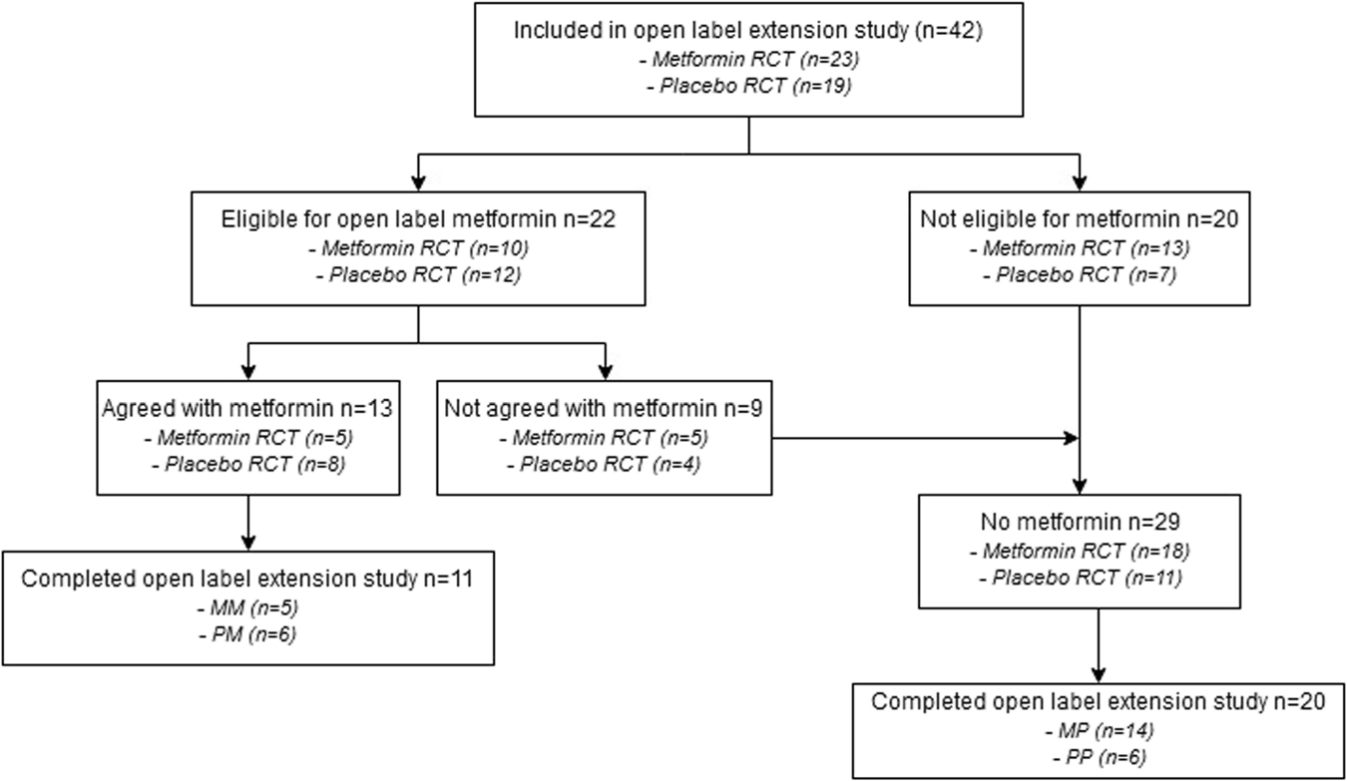
Flowchart of the study population. MM metformin during RCT and extension study, PM placebo during RCT and metformin during extension study, MP metformin during RCT and placebo during extension study, PP placebo during RCT and extension study

Figure 1 shows the flowchart of the study population. All 42 participants who completed the RCT were included for the open label extension study. At start of the study, 22/42 (52%) participants were eligible for metformin of which 13 (59%) agreed with treatment. The remaining participants 29 (69%) did not use metformin 5 (17.2%) without obesity, 15 (51.7%) without IR, and 9 (31.0%) without consent for treatment). Eleven participants were lost to follow-up during the study (i.e., 4MP, 5PP, 2PM); therefore, a total of 31 participants were analyzed.

### Baseline characteristics

The baseline characteristics of the participants who completed the open label extension study are presented in Table 1. A wide range was observed in demographic and laboratory data between all participants, and participants within the study-arms. Of the participants, 84% was obese and 45% had IR at start of the open label extension study. Study-arms differed significantly for HOMA-IR, but not for BMI or BMI-sds. Participants were predominantly female and in pubertal (Tanner 2–4) or postpubertal (Tanner 5) stages. The observed Tanner stages differed significantly between the study-arms.

**Table 1:** Baseline characteristics of participants who completed the open label extension study, stratified by study-arm (*n* = 31)

	All (*n* = 31)	MM (*n* = 5)	PM (*n* = 6)	MP (*n* = 14)	PP (*n* = 6)	*p*-Value
Demographics						
Age (years)	14.8 (11.6–17.9)	15.7 (14.8–17.6)	14.0 (12.5–16.6)	14.9 (11.7–17.9)	14.3 (11.6–17.8)	0.46
Sex male, *n* (%)	8 (26)	1 (20)	3 (50)	3 (21)	1 (17)	0.51
Height (cm)	168.3 (156.7–189.8)	171.0 (158.5–178.7)	167.6 (162.5–181.0)	166.8 (156.7–180.0)	166.0 (157.0–189.8)	0.64
Height-sds	0.1 (−1.6–2.7)	−0.1 (−1.2–0.9)	0.5 (−1.6–2.7)	0.0 (−1.1–0.8)	0.5 (−1.1–1.5)	0.65
Weight (kg)	88.4 (56.9–130.9)	94.2 (79.0–107.3)	95.9 (77.4–130.9)	80.3 (56.9–105.6)	78.3 (69.4–118.4)	0.17
BMI (kg/m^2^)	31.2 (22.3–45.1)	31.7 (30.6–34.4)	32.1 (28.4–45.1)	27.7 (22.3–41.4)	28.5 (25.9–44.9)	0.12
BMI-sds	2.9 (1.4–4.5)	3.1 (2.8–3.4)	3.4 (2.8–4.5)	2.5 (1.4–4.2)	2.9 (2.0–4.5)	0.14
Hip circumference (cm)	102.3 (82.5–138.0)	104.0 (99.5–112.0)	105.8 (93.5–127.0)	95.0 (82.5–119.0)	100.5 (91.5–138.0)	0.13
Waist circumference (cm)	100.6 (77.0–137.5)	100.0 (92.0–109.0)	109.8 (94.0–130.0)	94.0 (77.0–119.7)	97.5 (89.0–137.5)	0.23
SBP (mmHg)	118 (102–133)	125 (113–133)	125 (114–130)	115 (102–127)	113 (105–120)	**0.04**
DBP (mmHg)	69 (45–87)	74 (63–87)	77 (54–85)	67 (45–77)	69 (60–72)	0.21
Tanner stage, *n* (%)						**0.04**
Prepubertal	1(3)	0	0	1 (7)	0	
Pubertal	14 (45)	2 (40)	5 (83)	4 (29)	5 (83)	
Postpubertal	16 (52)	3 (60)	1 (17)	9 (64)	1 (17)	
Biochemical measurements						
Glucose (mmol/l)	4.7 (4.1–5.6)	4.6 (4.1–5.1)	5.0 (4.1–5.6)	4.6 (4.2–5.5)	4.7 (4.3–5.5)	0.79
Insulin (mmol/l)	16 (1–48)	20 (17–25)	24 (16–48)	11 (1–33)	12 (3–18)	**<0.01**
HOMA-IR	3.4 (0.2–8.8)	3.7 (3.6–5.1)	5.2 (3.6–8.8)	2.4 (0.2–6.2)	2.5 (0.7–3.4)	**<0.01**
HbA1c (mmol/mol)	34 (29–40)	30 (29–36)	37 (29–39)	34 (29–40)	35 (31–39)	0.31
Cholesterol (mmol/l)	4.5 (3.0–6.8)	4.7 (3.2–5.5)	3.9 (3.4–5.0)	4.2 (3.6–5.9)	5.4 (3.0–6.8)	0.38
HDL (mmol/l)	1.20 (0.85–1.67)	1.09 (1.02–1.37)	1.29 (0.93–1.48)	1.13 (0.85–1.67)	1.23 (0.96–1.44)	0.93
LDL (mmol/l)	2.7 (1.4–4.9)	2.8 (1.8–3.3)	2.3 (1.7–3.3)	2.6 (1.8–3.6)	3.4 (1.4–4.9)	0.23
TG (mmol/l)	1.2 (0.5–2.8)	1.1 (0.6–1.9)	1.1 (0.8–1.6)	1.1 (0.5–2.8)	1.1 (0.7–1.6)	0.99
ALAT (U/l)	21 (8–70)	20 (9–70)	17 (8–32)	15 (10–60)	16 (9–32)	0.92
Kreatinin (µmol/l)	56 (43–76)	63 (45–76)	57 (44–74)	53 (44–64)	52 (43–66)	0.23
Vitamin B12 pmol/l	299 (108–505)	263 (239–380)	337 (248–434)	298 (108–494)	225 (156–505)	0.65

Data presented as median with range or frequency with percentage. *p*-Value represents the differences between the four study-arms. Bold entries are used for *p*-values which were below the significance level of <0.05

*MM* metformin during RCT and open label extension study, *PM* placebo during RCT and metformin during open label extension study, *MP* metformin during RCT and placebo during open label extension study, *PP* placebo during RCT and open label extension study, *BMI* Body mass index, *sds* standard deviation score, *SBP* systolic blood pressure, *DBP* diastolic blood pressure, *HOMA-IR* Homeostatic Model Assessment for Insulin Resistance

### Effect on BMI and HOMA-IR

**Fig. 2:**
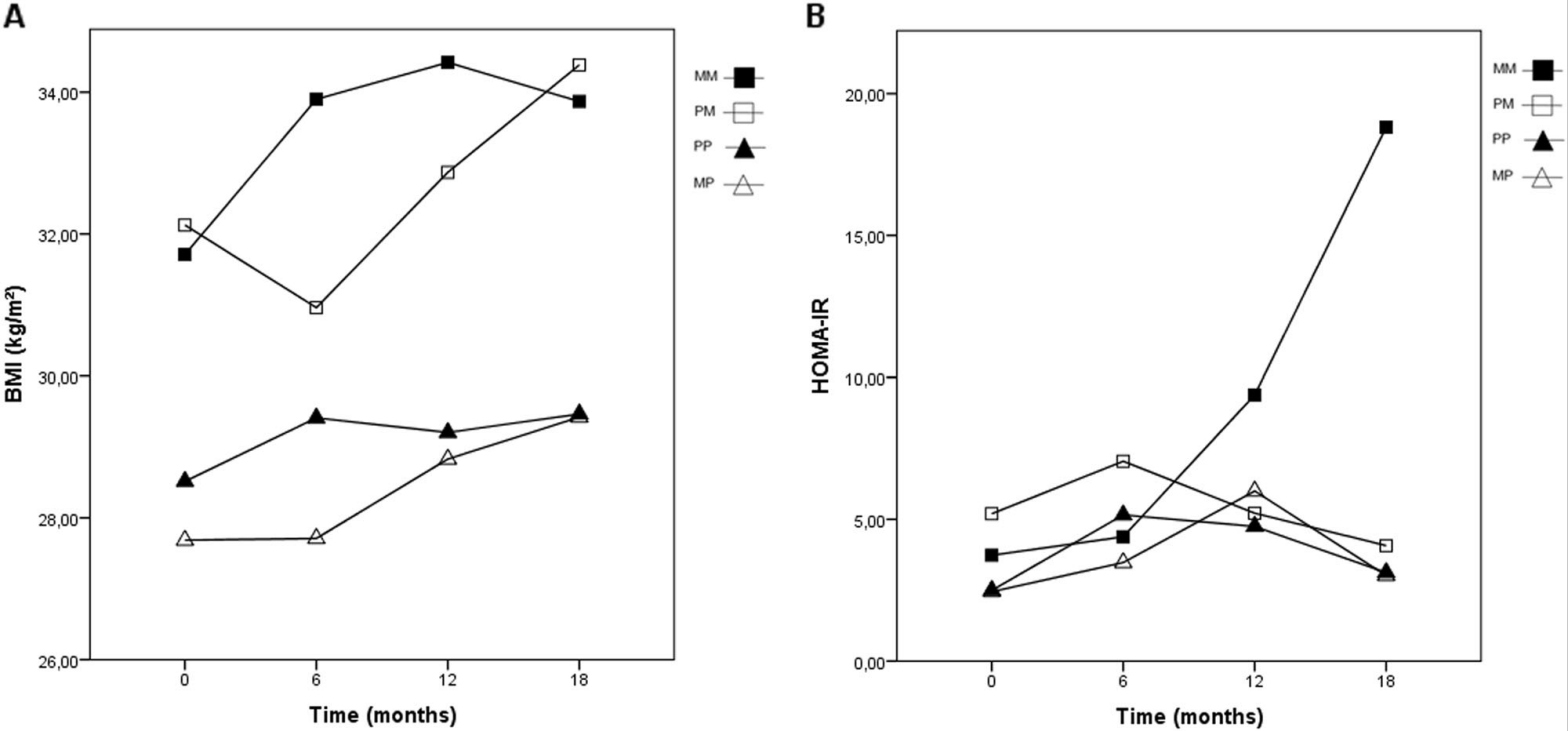
Progression of BMI and HOMA-IR over the open label extension study, stratified by study-arm. Median BMI (**a**); median HOMA-IR (**b**). MM metformin during RCT and extension study, PM placebo during RCT and metformin during extension study, PP placebo during RCT and extension study, MP metformin during RCT and placebo during extension study

Figure 2 shows the progression of the BMI and HOMA-IR during the open label extension study, stratified by study-arm. In the MM subgroup, an overall increase in BMI was observed from the start of the open label extension study. In the PM subgroup, an initial decrease in BMI was observed and thereafter an increase. In the MP subgroup, a stabilization of BMI was noticed in the first 6 months and thereafter an increase. In the PP subgroup, an initial increase in the first 6 months and thereafter a stabilization in BMI was observed. For HOMA-IR, a sharp increase was observed in the MM subgroup. In the other subgroups a wavy pattern was observed. An overview of the absolute values as well as the changes of the BMI, HOMA-IR, and BMI-sds over the open label extension study, RCT, and RCT and open label extension study together are presented in supplementary Table 1 and Supplementary Fig. 1.

### Safety and tolerability

No serious adverse effects were reported. Concerning safety measurements, liver dysfunction defined as an ALAT above safety limits was observed in 2 participants (1 MM and 1 MP). A low vitamin B12 was seen in 2 participants (1 MM and 1 MP). No renal impairment was observed. Metformin was generally well-tolerated. Two participants reported nausea and four diarrhea. Two participants (both PM) discontinued the study due to side effects (gastrointestinal symptoms).

### Other outcomes

**Fig. 3:**
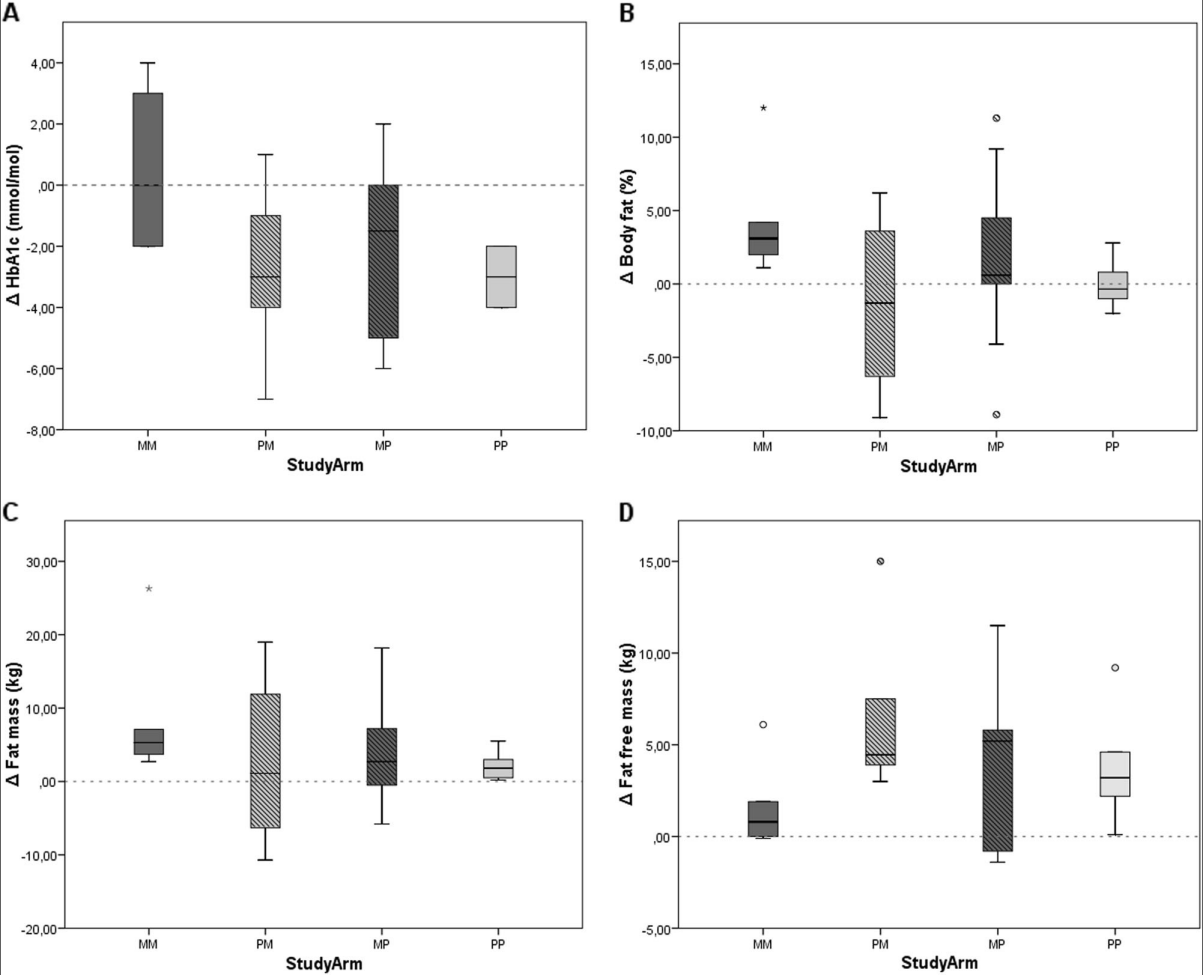
Change of HbA1c, body fat percentage, fat mass, and fat-free mass in the open label extension study. MM metformin during RCT and extension study, PM placebo during RCT and metformin during extension study, PP placebo during RCT and extension study, MP metformin during RCT and placebo during extension study. Dotted line represents no change

Figure 3 shows the median changes of HbA1c, body fat percentage, fat mass, and fat-free mass. An increase in HbA1c was observed in the MM subgroup, whereas it decreased in the other subgroups. For body fat percentage a stabilization was observed in the PM and PP subgroup, whereas the MM and MP subgroup showed an increase. The fat mass increased in the MM and PP subgroup, where in the MP and PM subgroup a median decrease was observed with a wide range. For fat-free mass, an increase was observed in all subgroups. An overview of the absolute values as well as the changes of the HbA1c, body fat percentage, fat mass, and fat-free mass over the open label extension study, and RCT and open label extension study together are presented in supplementary Table 1.

Reliable analysis regarding the change in physical fitness and quality of life could not be performed, since only a few participants performed the fitness test (*n* = 11) and/or handed in the IWQOL assessment (*n* = 19) at the end of the RCT and at the end of the open label extension study.

### Obesity-related complications

At the end of the open label extension study, a disturbed glucose tolerance was observed in 8/31 (26%) participants. One participant developed T2DM (confirmed with an additional oral glucose tolerance test), 3 IFG, and 4 IGT. High triglycerides were observed in 7/31 (23%) participants, and low HDL in 11/31 (36%). Furthermore, 2 participants developed a high systolic and/or diastolic blood pressure. Microalbuminuria was observed in 1/31 (3%) participant. In addition, in the entire population an increase in vascular stiffness measured with the AIx (−3.1 vs. 2.3; *p* = 0.04) was noticed, no significant difference was observed for PWV (4.2 vs. 4.6; *p* = 0.10). An overview of the progression to obesity-related metabolic and cardiovascular complications over the RCT and open label extension study is shown in Table 2.

**Table 2:** Progression to metabolic and cardiovascular complications of participants who completed the open label extension study (*n* = 31)

	Start RCT *T* = 0	End RCT *T* = 18	End extension study *T* = 36	*p*-Value T0 vs. T18	*p*-Value T18 vs. T36	*p*-Value T0 vs. T36
Obese waist	30 (97)	25 (81)	26 (84)	**0.03**	1.00	**0.03**
Impaired FPG	—	1 (3)	3 (10)	0.37	0.53	0.15
IGT	2 (6)	2 (6)	4 (13)	0.41	1.00	0.68
T2DM	—	—	1 (3)	—	—	—
High triglyceride	8 (26)	6 (19)	7 (23)	0.53	0.71	0.74
Low HDL	6 (19)	8 (26)	11 (36)	0.48	0.26	0.06
Microalbuminuria	1 (3)	1 (3)	1 (3)	—	—	—
High SBP	8 (26)	7 (23)	2 (6)	1	1	**0.03**
High DBP	2 (6)	3 (10)	1 (3)	0.56	0.56	0.56
MetS	7 (23)	6 (19)	9 (29)	0.74	0.26	0.56
AIX (%)	−0.2 (−19.0–17.5)	−3.1 (−29.0–16.5)	2.3 (−19.0–20.0)	0.12	**0.04**	0.25
PWV (ms-1)	3.9 (2.4–5.0)	4.2 (1.5–6.4)	4.6 (3.1–6.7)	0.10	0.10	**<0.01**
PWV-sds	0.3 (0.2–0.7)	0.4 (0.2–7)	0.4 (0.2–0.6)	0.31	0.62	0.14

*p*-Values represents the differences between follow-up measurements in study participants (Wilcoxon signed rank test). Bold entries are used for *p*-values which were below the significance level of <0.05.

*RCT* randomized controlled trail, *FPG* impaired fasting plasma glucose, *IGT* impaired glucose tolerance, *T2DM* type 2 diabetes mellitus, *HDL* high-density lipoprotein, *SBP* systolic blood pressure, *DBP* diastolic blood pressure, *MetS* metabolic syndrome, *AIX* augmentation index, *PWV* pulse wave velocity

## Discussion

In this study, adolescents were participating in an 18 month open label extension study following an 18 month double-blinded RCT on the long-term efficacy, safety, and tolerability of metformin in adolescents with obesity and IR^22^. At start of the open label extension study, metformin was offered to participants depending on BMI and HOMA-IR; therefore, 4 study-arms were created in which participants used respectively 18 months, 36 months, or no metformin at all.

Short-term beneficial effects of metformin have been described previously, resulting in a decrease in BMI ranging between −0.2 to −2.1 kg/m^2^ after maximal 6 months of treatment^33^. Studies on the long-term effect (>6 months) of metformin on BMI are limited^21–24^. In these studies, the maximum decrease of BMI was achieved after 6–12 months of treatment, after which the BMI stabilized or slightly increased^21–24^. In the current study—in accordance with these reports—an initial decrease in BMI was noticed in participants who started with metformin treatment till 6 months of follow-up. The patients continuing metformin treatment however showed an increase in BMI. Our results suggest that the BMI continues to increase despite prolonged metformin treatment. This finding is in contrast with the long-term effects of metformin in non-diabetic adults, in which metformin treatment resulted in significant more weight loss in comparison with placebo after 2.8 years and even after 10 years^34,35^. In adults, it has been described that compliance with therapy is a major determinant for the effectiveness of metformin^25^. In contrast to the RCT, no pill counts were performed in the open label extension study, so the actual compliance to therapy could not be monitored. However, some participants reported not to take the metformin tablets on daily basis, which implies under-treatment. This could be an explanation why the ongoing improvement on BMI was not observed in our study. Apart from compliance leading to under-treatment, the maximum used metformin dosage per day could have influenced the effect of long-term metformin treatment. The maximum recommended dosage in adolescents is currently 2000 mg/day, which was also prescribed to our participants^36^. In adults the maximum dose is 3000 mg/day. It is known that the clearance of many drugs is higher in adults with obesity compared to healthy weight adults^37^. In the RCT a pharmacokinetic study was performed, which showed that the clearance of metformin in adolescents with obesity is comparable to adults without obesity (unpublished). This indicates that the maximum daily doses of metformin in obese adolescents could safely be raised up to the adult dosage. This is relevant since results of a recent study suggests that the effect of metformin could be dosage dependent^20^. The continuation of weight gain during metformin treatment observed in some of the participants in our study might therefore be caused by an insufficient dosage, leading to under-treatment, especially since most of the “non-responders” weighted >100 kg. Since only minor side effects (i.e., nausea and diarrhea) were observed during the first weeks of treatment, it can be assumed that a higher dosage will be tolerated as well.

For HOMA-IR, a sharp increase was noticed in the MM subgroup (36 months metformin) from the start of the open label extension study onwards. Further exploration revealed that two participants showed a sharp increase in HOMA-IR. This could be explained by the lack of compliance to metformin therapy or to an insufficient dosage as previously described, both leading to under-treatment. On the other hand, a high intra-individual variation in insulin concentration up to 12% in combination with a relatively low reproducibility in insulin measurements, both affecting HOMA-IR should be taken into account^38,39^. This might also explain the wavy pattern of HOMA-IR observed in the other subgroups. The difference between the MM subgroup and the others could not be fully elucidated; however, the sharp increase in HOMA-IR might possibly be the first sign towards development of T2DM. Metformin seems not to have major effects on HOMA-IR, although the PM subgroup decreased slightly more than the MP and PP subgroup, which is in line with literature^16,17,21,22^. Since IR is known to be associated with BMI, the increasing BMI in some participants might possibly have influenced the results^5,30^. Furthermore, pubertal stage may have influenced the HOMA-IR as most of the participants were postpubertal at the end of current study. The observed decrease in HOMA-IR might therefore be related to the physiological postpubertal decrease of IR, which occurs after an initial increase of IR during puberty due to high circulating levels of growth hormone^40^. Despite these limitations, insulin is still considered as the best available screenings method for IR in daily clinical practise^30,39^.

Since the prevalence of (pediatric) obesity is still increasing^2^, and consequently obesity-related metabolic and cardiovascular complications, studies into additional treatment options are of great importance. In the current study, obesity-related complications were already observed at start of the RCT in some participants, and the number had increased after 36 months of follow-up. The protective effect of metformin on the development of obesity-related complications could not be demonstrated, since most participants (25/31) used metformin during the RCT and/or open label extension study. Several others studied the short-term effect of metformin on obesity-related complications and showed in most cases a positive effect on FPG, but not on lipids and/or blood pressure^15–18,21,33^. Although the protective effect of metformin on the development of complications, especially T2DM, could not be demonstrated in previously performed pediatric studies nor in the current study, however results in adults are promising. It has been shown in adults that metformin decreases the cumulative incidence of T2DM over a follow-up period up to 15 years^34,35,41^. Despite the lower T2DM incidence, microvascular complications were not less frequently observed in patients with metformin treatment after an average follow-up period of 15 years^41^. However, subjects who developed T2DM had significantly more microvascular complications in comparison with those without T2DM^41^. Although a sustained effect of long-term use of metformin could not be demonstrated in the current study, it seems reasonable, taken the promising results of adults studies into account, to recommend off-label metformin in addition to lifestyle intervention in adolescents with obesity to manage weight loss and to prevent/delay the development of T2DM. Though, further studies on the long-term efficacy of metformin in children/adolescents with obesity are warranted.

To the best of authors knowledge, this is the first study to report on the efficacy, safety, and tolerability of metformin in adolescents with obesity and IR with a follow-up period of 36 months. However, certain limitations must be mentioned. Firstly, the number of study participants included was lower than anticipated. This might be a consequence of the studied population, which were teenagers often from from ethnic minorities and/or low-income families who were hard to recruit and retain in the study^42,43^. Secondly, due to the design of this open label extension study participants were divided into four study-arms and therefore limiting the number of participants per arm. Due to previous mentioned limitations and not normally distributed data, analysis was restricted. Therefore, analysis taken potential confounders such as sex and Tanner stage into account could not be performed. Relatively few participants used metformin during the open label extension study which could be caused by the start/continuation criteria for metformin, as 20/42 (48%) participants did not qualify for metformin therapy as they did not suffer from obesity and/or IR anymore. On the other hand, this might be caused by limited motivation to use metformin, which is reflected by the fact that 9/22 (41%) participants eligible for metformin did not agree with therapy. In addition, 7/11 (64%) dropouts were participants who were entitled for metformin therapy.

The high dropout is in concordance with other obesity studies, irrespectively of study duration^15,19,21,23,24^, and also observed during routine clinical care at pediatric (obesity) outpatient clinics. Since studies in populations with obesity are limited by the number of inclusions and high dropouts, it is suggested that for RCT’s less restrictive inclusion/exclusion criteria should be used in combination with the formation of research networks to accomplish adequate inclusion numbers^42^. When considering studies in pediatric populations with obesity it is questionable whether they should be performed in RCT’s or whether it is more practical to perform studies in a daily clinical care setting using a standardized protocol.

## Conclusion

While metformin treatment in metformin naive participants seems to result in an initial decreases in BMI and HOMA-IR, there is no evidence for sustained effect after prolonged use in adolescents. Limited compliance and/or insufficient dose may explain the differences in long-term effects between adolescents and adults.

## Electronic supplementary material

Supplemantary Table 1

Supplemenatry Table 2

Supplemenatry Figure 1
