# Supplementary material for: Long-term metformin treatment in adolescents with obesity and insulin resistance, results of an open label extension study
Source: Nutr Diabetes. 2018 Sep 10;8:47. doi: 10.1038/s41387-018-0057-6 (PMC6129504; doi:10.1038/s41387-018-0057-6)
Supplement: Supplementary file 1 — Supplemantary Table 1 [file 41387_2018_57_MOESM1_ESM.docx]

**Supplemental Table 1. Baseline characteristics at start of the RCT of participants who completed the open label extension study, stratified by study-arm n=31**

|  | **All (n=31)** | **MM (n=5)** | **PM (n=6)** | **MP (n=14)** | **PP (n=6)** | **p-value** |
| --- | --- | --- | --- | --- | --- | --- |
| *Demographics* | |  |  |  |  |  |
| **Age (years)** | 13.3 (10.2-16.5) | 14.2 (13.4-16.2) | 12.6 (10.9-15.2) | 13.5 (10.2-16.5) | 13.5 (10.2-16.3) | 0.420 |
| **Sex male n(%)** | 8 (25.8) | 1 (20) | 3 (50) | 3 (21.4) | 1 (16.7) | 0.508 |
| **Height (cm)** | 163.6 (150.1-179.2) | 171.0 (154.3-179.2) | 164.5 (157.0-171.0) | 162.1 (150.1-169.0) | 161.2 (151.8-177.8) | 0.356 |
| **Height-sds** | 0.22 (-1.3-2.9) | 0.2 (-1.3-1.1) | 0.5 (-1.2-2.9) | 0.1 (-0.9-1.0) | 0.5 (-1.0-0.9) | 0.836 |
| **Weight (kg)** | 84.4 (58.6-117.8) | 89.8 (82.2-113.3) | 88.3 (74.0-117.8) | 79.3 (58.6-98.9) | 70.5 (65.0-115.7) | 0.196 |
| **BMI (kg/m²)** | 31.4 (25.3-44.1) | 32.3 (29.3-35.3) | 32.2 (30.0-41.7) | 28.9 (25.3-38.1) | 29.5 (25.4-44.1) | 0.130 |
| **BMI-sds** | 3.2 (2.4-4.4) | 3.3 (2.7-3.7) | 3.7 (3.3-4.3) | 3.0 (2.4-3.9) | 3.1 (2.4-4.4) | 0.157 |
| **Hip circumference (cm)** | 103.2 (87.2-132.0) | 105.0 (96.0-111.0) | 106.5 (95.0-123.1) | 101.0 (87.2-118.5) | 99.0 (94.5-132.0) | 0.236 |
| **Waist circumference (cm)** | 103.4 (88.5-128.0) | 97.5 (94.0-119.5) | 107.5 (100.0-124.0) | 97.5 (88.5-119.0) | 102.4 (89.0-128.0) | 0.552 |
| **SBP (mmHg)** | 118 (92-135) | 121 (115-134) | 123 (109-133) | 118 (105-135) | 109 (92-117) | 0.133 |
| **DBP (mmHg)** | 67 (46-85) | 72 (63-85) | 72 (55-83) | 69 (46-76) | 62 (52-65) | 0.149 |
| **Tanner stage**  **-prepubertal**  **-pubertal**  **-postpubertal** | 6 (19.4)  18 (58.1)  6 (19.4) | -  3 (60)  2 (40) | 2 (33.3)  3 (50.0)  1 (16.7) | 3 (21.4)  10 (71.4)  1 (7.1) | 1 (16.7)  2 (50.0)  2 (33.3) | 0.848 |
| *Biochemical measurements* | |  |  |  |  |  |
| **Glucose (mmol/l)** | 4.8 (4.3-5.5) | 4.8 (4.7-5.0) | 4.8 (4.4-5.2) | 4.8 (4.3-5.5) | 4.8 (4.5-4.9) | 0.555 |
| **Insulin (mmol/l)** | 21(1-47) | 21 (4-38) | 24 (6-41) | 18 (1-47) | 16 (11-25) | 0.503 |
| **HOMA-IR** | 4.6 (0.2-9.8) | 4.4 (0.9-8.1) | 4.9 (1.2-9.1) | 4.0 (0.2-9.8) | 3.3 (2.4-5.3) | 0.547 |
| **HbA1c (mmol/mol)** | 32 (25-39) | 33 (27-37) | 32 (28-34) | 33 (25-39) | 32 (29-34) | 0.518 |
| **Cholesterol (mmol/l)** | 4.6 (2.8-7.8) | 5.3 (3.2-5.4) | 4.2 (3.7-7.8) | 4.5 (3.7-6.1) | 4.4 (2.8-5.1) | 0.574 |
| **HDL (mmol/l)** | 1.19 (0.80-1.77) | 1.16 (0.90-1.45) | 1.15 (1.10-1.53) | 1.10 (0.80-1.77) | 1.31 (0.94-1.74) | 0.269 |
| **LDL (mmol/l)** | 2.8 (1.6-5.8) | 3.1 (1.6-3.8) | 2.35 (1.8-5.8) | 2.9 (1.8-4.3) | 2.4 (1.6-3.2) | 0.656 |
| **TG (mmol/l)** | 1.4 (0.5-3.4) | 1.3 (0.8-3.4) | 1.5 (0.7-1.9) | 1.5 (0.5-2.0) | 0.7 (0.6-1.4) | 0.538 |
| **ALAT (U/l)** | 23 (12-69) | 18 (12-69) | 21.5 (15-52) | 19.5 (12-42) | 13 (12-48) | 0.242 |
| **Kreatinin (µmol/l)** | 54 (42-82) | 61 (51-82) | 54 (47-81) | 49.5 (42-55) | 48 (42-53) | **0.041** |
| **Vitamin B12 pmol/l** | 367 (190-617) | 374 (241-454) | 393 (256-495) | 368 (109-617) | 308 (223-543) | 0.949 |

*Data are presented as median with range, or frequency with percentage. RCT = randomized controlled trail, MM = metformin during RCT and open label extension study, PM = placebo during RCT and metformin during open label extension study, MP = metformin during RCT and placebo during open label extension study, PP = placebo during RCT and open label extension study BMI= Body mass index, sds = standard deviation score, SBP = systolic blood pressure, DBP = diastolic blood pressure. HOMA-IR = Homeostatic Model Assessment for Insulin Resistance. p-value represents the differences between the four study-arms. Bold entries are used for p-values which were below the significance level of <0.05.*
