# Supplementary material for: Long-term metformin treatment in adolescents with obesity and insulin resistance, results of an open label extension study
Source: Nutr Diabetes. 2018 Sep 10;8:47. doi: 10.1038/s41387-018-0057-6 (PMC6129504; doi:10.1038/s41387-018-0057-6)
Supplement: Supplementary file 2 — Supplemenatry Table 2 [file 41387_2018_57_MOESM2_ESM.docx]

**Supplemental Table 2. Treatment effects of metformin for the 4 study-arms of participant who completed the open label extension study n=31**

|  | **Study**  **arm** | **T0 (start RCT)** | **T18 (end RCT)** | **T36 (end OLEs)** | **Δ18_1_ (T18-T0)** | **p-value** | **Δ18_2_ (T36-T18)** | **p-value** | **Δ36 (T36-T0)** | **P-value** |
| --- | --- | --- | --- | --- | --- | --- | --- | --- | --- | --- |
| **BMI (kg/m²)** | MM  MP  PM  PP | 32.3 (29.3-35.3)  28.9 (25.3-38.1)  32.2 (30.0-41.7)  29.5 (25.4-44.1) | 31.7 (30.6-34.4)  27.7 (22.3-41.4)  32.1 (28.4-45.1)  28.5 (25.9-44.9) | 33.9 (31.7-42.6)  29.4 (22.9-48-4)  34.4 (29.9-45.2)  29.5 (26.4-48.0) | 1.3 (-3.1-2.0)  -1.4 (-4.5-3.3)  0.8 (-1.8-3.4)  1.00 (-1.2-2.4) | 0.161 | 2.2 (0.2-9.0)  1.9 (-1.8-8.5)  0.5 (-2.1-5.1)  1.1 (0.5-3.1) | 0.886 | 3.4 (-2.9-7.3)  -0.6 (-4.1-10.3)  1.3 (-0.6-6.4)  2.9 (-0.7-3.9) | 0.716 |
| **BMI-sds** | MM  MP  PM  PP | 3.3 (2.7-3.7)  3.0 (2.4-3.9)  3.7 (3.3-4.3)  3.1 (2.4-4.4) | 3.1 (3.8-3.4)  2.5 (1.4-4.2)  3.4 (2.8-4.5)  2.9 (2.0-4.5) | 3.3 (3.0-4.3)  2.9 (1.1-4.7)  3.6 (2.8-4.5)  2.9 (2.1-4.8) | 0.1 (-0.5-0.2)  -0.3 (-1.3-0.2)  -0.2 (-0.4-0.2)  0.1 (-0.5-0.2) | 0.241 | 0.2 (-0.1-0.9)  0.3 (-0.5-1.5)  -0.1 (-0.3-0.6)  0.1 (-0.1-0.3) | 0.761 | 0.3 (-0.6-0.6)  -0.2 (-1.4-0.7)  -0.2 (-0.4-0.3)  0.1 (-0.4-0.4) | 0.619 |
| **Body fat %** | MM  MP  PM  PP | 36.7 (39.0-51.0)  38.3 (30.8-49.2)  41.9 (40.9-54.7)  36.7 (33.7-51.0) | 39.9 (29.7-43.1)  35.2 (26.1-47.1)  42.7 (38.5-50.4)  37.7 (29.8-51.9) | 43.0 (40.7-45.1)  36.0 (28.0-49.4)  38.9 (38.0-54.0)  37.4 (27.8-52.7) | -0.2 (-6.1-2.8)  -3.6 (-10.2-4.0)  -1.8 (-4.3-7.3)  -0.5 (-3.9-2.1) | 0.506 | 3.1 (1.1-12.0)  0.6 (-8.9-11.3)  -1.3 (-9.1-6.2)  -0.35 (-2.0-2.8) | 0.137 | 3.2 (-3.7-7.0)  -1.0 (-9.6-5.6)  -2.3 (-4.7-3.5)  -0.1 (-5.9-1.7) | 0.336 |
| **Fat mass (kg)** | MM  MP  PM  PP | 26.8 (22.9-58.6)  29.5 (18.6-45.2)  36.5 (30.3-62.2)  26.8 (22.9-58.6) | 34.8 (31.1-42.8)  29.1 (15.5-54.3)  42.7 (29.6-65.4)  29.5 (22.6-60.9) | 44.6 (33.8-57.9)  31.7 (21.2-55.2)  44.8 (32.5-84.4)  31.3 (25.6-66.4) | 2 (-9.5-4.4)  -0.5 (-7.2-25.0)  2.5 (-3.5-14.5)  2.1 (-2.5-6.9) | 0.375 | 5.3 (1.1-12.0)  2.7 (-5.8-18.2)  1.1 (-10.7-19.1)  1.8 (0.2-5.5) | 0.463 | 7.3 (-2.5-16.8)  3.3 (-8.5-17.7)  5.2 (-3.6-22.2)  4.9 (0.5-8.9) | 0.726 |
| **Fat free mass (kg)** | MM  MP  PM  PP | 42.8 (39.0-61.5)  48.0 (36.1-54.9)  50.4 (42.8-66.6)  42.8 (39.0-61.5) | 56.2 (47.4-74.8)  49.2 (40.3-61.6)  52.5 (47.3-71.3)  48.6 (42.4-77.9) | 56.2 (49.3-80.9)  51.0 (44.7-67.1)  62.3 (51.6-75.2)  52.6 (44.6-87.1) | 2 (0.3-3.5)  1.2 (3.10-12.2)  1.4 (0.5-12.8)  5.5 (0.1-16.4) | 0.247 | 0.8 (-0.1-6.1)  5.2 (-1.4-11.5)  4.5 (3.0-15.0)  3.2 (0.1-9.2) | 0.363 | 3.9 (0.3-9.3)  8.3 (-3.8-17.7)  8.8 (3.5-20.3)  8.8 (2.1-25.6) | 0.335 |
| **HOMA-IR** | MM  MP  PM  PP | 4.4 (0.9-8.1)  4.0 (0.2-9.8)  4.9 (1.2-9.1)  3.3 (2.4-5.3) | 3.7 (3.6-5.1)  2.5 (0.2-6.2)  3.2 (3.6-8.8)  2.5 (0.7-3.4) | 18.8 (3.2-52.1)  3.0 (0.8-31.9)  4.1 (3.1-7.0)  3.1 (0.2-3.8) | -0.7(-3.0-2.7)  -2.0(-7.3-11.4)  -0.6(-3.5-7.6)  -0.9(-3.8-3.1) | 0.383 | 13.7 (1.6-48.3)  1.2 (-5.4-29.4)  -1.1 (-4.6-1.4)  0.4 (-0.8-2.2) | 0.137 | 10.7 (0.2-47.0)  0.5(-9.1-22.8)  -1.4(-2.3-3.0)  -0.4(-2.2-2.3) | 0.052 |
| **HbA1c (mmol/mol)** | MM  MP  PM  PP | 33 (27-37)  33 (25-39)  32 (28-34)  32 (29-34) | 30 (29-36)  34 (29-40)  37 (29-39)  35 (31.39) | 34 (27-34)  31.5 (26-42)  32.5 (28-35)  32 (29-35) | -1 (-4-2)  1 (-6-6)  4 (-2-8)  4.5 (-2-5) | 0.075 | 0 (-2-4)  -1.5 (-6-2)  -3 (-7-1)  -3 (-4- -2) | 0.150 | 0 (-3-2)  -2.0 (-4-3)  0.5 (-1-3)  1 (-4-2) | 0.336 |

*Data are presented as median with range. RCT = randomized controlled trail, MM = metformin during RCT and open label extension study, PM = placebo during RCT and metformin during open label extension study, MP = metformin during RCT and placebo during open label extension study, PP = placebo during RCT and open label extension study BMI= Body mass index, sds = standard deviation score, HOMA-IR = Homeostatic Model Assessment for Insulin Resistance. P-values for differences in ΔT=18-T=0, ΔT=36-T=18, ΔT=36-T=0, between the 4 study-arm*
