# Supplementary material for: Long-term metformin treatment in adolescents with obesity and insulin resistance, results of an open label extension study
Source: Nutr Diabetes. 2018 Sep 10;8:47. doi: 10.1038/s41387-018-0057-6 (PMC6129504; doi:10.1038/s41387-018-0057-6)
Supplement: Supplementary file 3 — Supplemenatry Figure 1 [file 41387_2018_57_MOESM3_ESM.docx]

**Supplementary Figure 1. Progression of BMI and HOMA-IR over the RCT and open label extension study together, stratified by study-arm. median BMI (a); median HOMA-IR (b).**


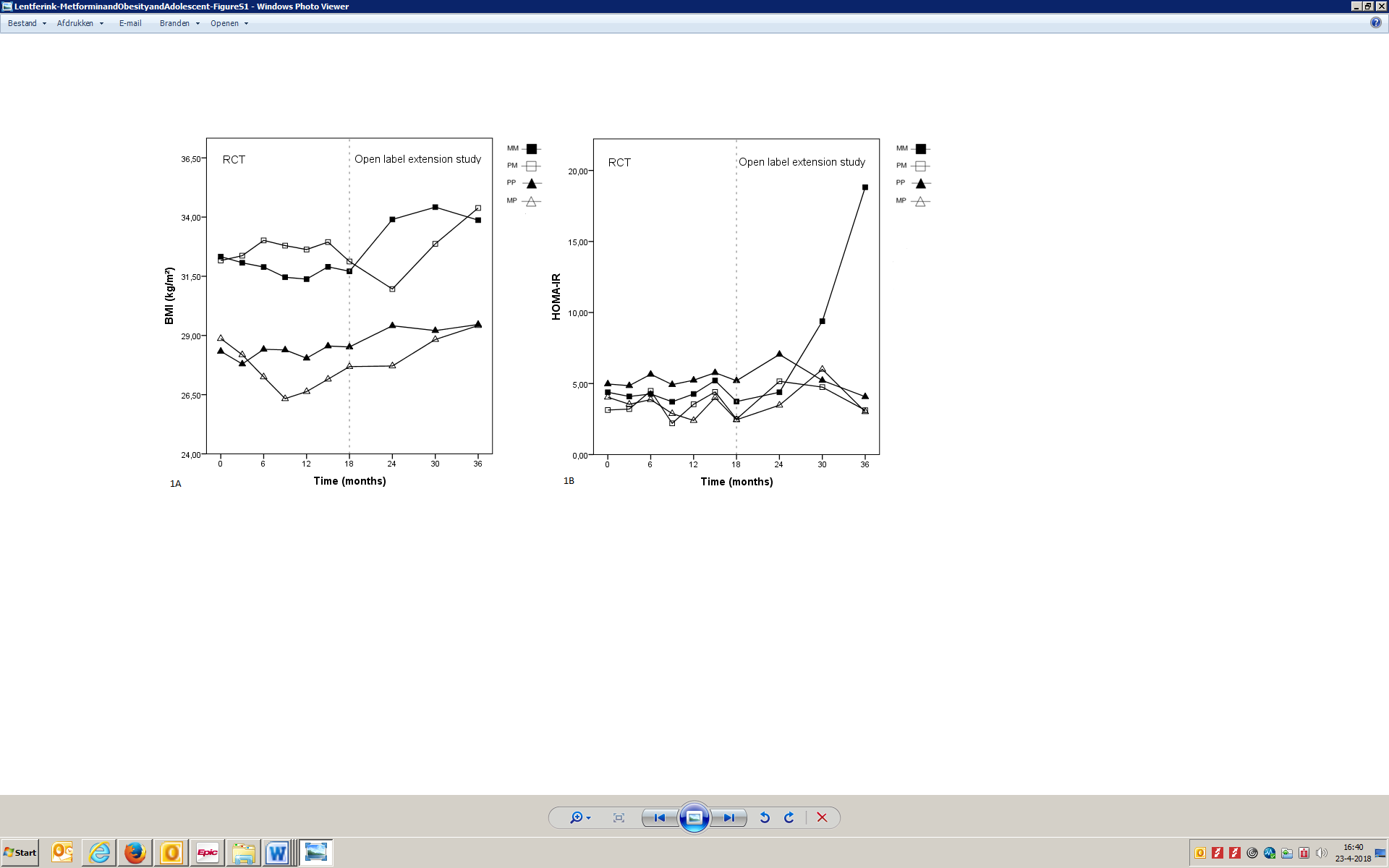


*MM = metformin during RCT and extension study, PM = placebo during RCT and metformin during extension study, PP = placebo during RCT and extension study, MP = metformin during RCT and placebo during extension study.*
